# Functional brain alterations in auditory hallucination subtypes in individuals with auditory hallucinations without the diagnosis of specific neurological diseases and mental disorders at the current stage

**DOI:** 10.1002/brb3.1487

**Published:** 2019-11-29

**Authors:** Xiaodong Lin, Chuanjun Zhuo, Gongying Li, Jie Li, Xiangyang Gao, Ce Chen, Deguo Jiang

**Affiliations:** ^1^ Department of Psychiatry Wenzhou Seventh People's Hospital Wenzhou China; ^2^ Department of Psychiatry‐Brian Micro and Macro Imaging Centre Department of Psychiatry School of Mental Health Jining Medical University Jining China; ^3^ Psychiatric‐Neuroimging‐Genetics and Comorbidity Laboratory (PNGC‐Lab) Tianjin Mental Health Center Tianjin Anding Hospital Tianjin China; ^4^ Center for Health Statistics Big Data Center of Chronic Disease Health Management Institute 301 Hospital of Chinese People's Liberation Army General Hospital of Chinese People's Liberation Army Beijing China

**Keywords:** auditory verbal hallucination, brain functional alterations, hypothesis of auditory hallucinations, subtypes of auditory hallucinations

## Abstract

**Background:**

We explored common and distinct pathological features of different subtypes of auditory hallucinations (AHs) to elucidate the underlying pathological mechanisms.

**Methods:**

We recruited 39 individuals with constant commanding and commenting auditory verbal hallucinations (CCCAVHs), 49 with own thought auditory verbal hallucinations (OTAVHs), 46 with nonverbal AHs (NVAHs), 32 with replay AVHs (RAVHs), and 50 healthy controls. Functional connectivity density mapping was used to investigate global functional connectivity density (gFCD) alterations in these AH groups relative to the control group.

**Results:**

We observed common brain functional alterations among four subtypes of AHs, such as increased gFCD in the bilateral superior temporal gyrus and mesial frontal lobe, and decreased gFCD in the bilateral medial prefrontal cortex. Increased gFCD was detected in the bilateral insula in CCCAVH individuals, bilateral thalamus in OTAVH individuals, bilateral precuneus in NVAH individuals, and bilateral hippocampus in RAVH individuals. The common and distinct gFCD alterations among four AH subtypes were located in main components of the frontoparietal, default mode, salience, central executive, and memory networks. Different AH subtypes exhibited specific aberrant patterns.

**Conclusions:**

Our findings suggest that aberrant functional activity and metabolism in the abovementioned networks play key roles in the occurrence of AHs. Our findings provide evidence for distinct gFCD alterations in specific AH subtypes.

## Graphical abstract

Different subtypes of auditory hallucinations (AHs) were underpinned by distinct functional brain activity patterns. The common and distinct gFCD alterations among these four AHs subtypes were observed mainly in components of the frontoparietal network, default mode network, salience network, central executive network, and memory network. Aberrant functional activity and metabolism in the abovementioned networks play key roles in the occurrence of AHs.

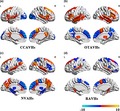

## 1 INTRODUCTION

Auditory hallucinations (AHs) are psychotic symptoms characterized by the lack of corresponding external stimuli. Individuals experience vivid perceptions of sound that have a strong sense of reality. The prevalence of AHs in patients with schizophrenia is approximately 70% (Blom, 2015; Maijer, Begemann, Palmen, Leucht, & Sommer, 2018). AHs also occur in other psychiatric disorders and neurological diseases. AHs may also occur in the general population, with a reported prevalence of 10%–15% (Alderson‐Day et al., 2016; Daalman, Diederen, Hoekema, Lutterveld, & Sommer, 2016; Diederen, Daalman, et al., 2012; Diederen, van Lutterveld, & Sommer, 2012; Garrison et al., 2017; Sommer et al., 2010). AHs are associated with a high incidence of self‐harm, aggression, and increased suicide risk in individuals that experience them, especially during adolescence and early adulthood (Brennan, Mednick, & Hodgkin, 2000; Fujita et al., 2015; Harris & Barraclough, 1997; Hodelet, 2001; Slotema et al., 2017). Nevertheless, the neural mechanisms of AHs have not been fully elucidated. As such, effective intervention strategies for persistent AHs are lacking, with poor therapeutic outcomes for AHs (Breier, Schreiber, Dyer, & Pickar, 1991; Craig et al., 2018; Craig, Ward, & Rus‐Calafell, 2016; Harkavy‐Friedman et al., 2003; Johns et al., 2014; Lee, Choi, Song, Chung, & Suh, 2004; Shergill, Murray, & McGuire, 1998; Thomas et al., 2019). From 2012, The International Consortium on Hallucination Research (ICHR) advocated that pathological features of AHs and effective treatment strategies should be investigated from multiple perspectives (Humpston et al., 2019; Jardri, Larøi, Waters, & International Consortium on Hallucination Research, 2019; Waters, 2012; Waters, Aleman, Fernyhough, & Allen, 2012; Waters, Woods, & Fernyhough, 2014).

Auditory hallucinations are observed in neurological diseases (such as dementia, Parkinson's disease, tinnitus, epilepsy, and other neurodegenerative conditions) and mental disorders (such as schizophrenia, schizoaffective disorder, major depression disorder, bipolar disorder, post‐traumatic stress disorder, anxiety disorder, borderline personality disorder, alcohol withdrawal, and narcolepsy), as well as in the general populations. According to previous studies, the lifetime prevalence of AHs in the general population is between 7.3% and 13.2% (Beavan, Read, & Cartwright, 2011; Connell et al., 2019; Kråkvik et al., 2015; Larøi, 2012; Larøi et al., 2019). Individuals without neurological diseases and mental disorders that experience AHs are termed “healthy individuals with auditory verbal hallucinations (Hi‐AVHs),” “voice‐hearers in the general population,” nonpatient voice‐hearers, hallucinatory experiencers without mental illness, and/or nonpsychotic adults with frequent auditory verbal hallucinations (Beavan et al., 2011; Connell et al., 2019; Daalman et al., 2016; Diederen, Daalman, et al., 2012; Diederen, van Lutterveld, et al., 2012; Larøi, 2012; Larøi et al., 2019; Sommer et al., 2010). The field suggests that these different terminologies should be standardized to provide clear criteria for classification; for example, many scholars have indicated that Hi‐AVHs may have pathological experiences and discomfort from persistent auditory verbal hallucinations (Beavan et al., 2011; Connell et al., 2019; Daalman et al., 2016; Diederen, Daalman, et al., 2012; Diederen, van Lutterveld, et al., 2012; Larøi, 2012; Larøi et al., 2019; Sommer et al., 2010); these individuals are at high risk of developing psychosis or other mental disorders. Hence, in this study, we defined our sample as auditory hallucination experiencers without specific neurological diseases or mental disorders in their current stage (i.e., they did not satisfy diagnostic criteria of any type of disease or mental disorder according to current clinical and neurological manifestations). This sample was abbreviated as AVs‐NSNMCS individuals. AVs‐NSNMCS individuals were classified into four subtypes as follows: The first subtype was termed constant commanding and commenting auditory verbal hallucinations (CCCAVHs). The main clinical features of this subtype of AHs were repetitiveness, commands, first‐ or third‐person voices, and running commentaries, which were constantly experienced. The second subtype, termed own thought auditory verbal hallucinations (OTAVHs), featured a “heard voice” that was an individual's own voice/thoughts. The third subtype, known as nonverbal AHs (NVAHs), had main clinical features of hearing only single or some words that did not form sentences. The fourth subtype, termed replay auditory verbal hallucinations (RAVHs), had main clinical features of hearing a “voice” of heard speech (McCarthy‐Jones et al., 2014).

Previous studies have proposed that AVHs/AHs may be caused by disturbances in brain circuits and networks (Alderson‐Day et al., 2016, 2015; Alderson‐Day, McCarthy‐Jones, & Fernyhough, 2015; Allen, Larøi, McGuire, & Aleman, 2008; Allen et al., 2008; Benetti et al., 2015; Diederen, Daalman, et al., 2012; Diederen, van Lutterveld, et al., 2012; Hugdahl, 2015; McCarthy‐Jones et al., 2014; Upthegrove et al., 2016). Based on these studies, hypotheses for AVHs/AHs have been put forth, such as the unstable memories hypothesis, source‐monitoring hypothesis, interhemispheric miscommunication hypothesis, top‐down effect and bottom‐up predictions hypothesis, and hybrid models of AVH hypothesis. Although each hypothesis is supported by neuroimaging evidence and can explain certain subtypes of AHs to a degree, none of them is sufficient to fully explain all AHs. These hypotheses indicate that AHs are highly complex psychotic symptoms; hence, common and different pathological features of different AH subtypes should be explored to provide insight into the mechanisms underlying AHs (Allen et al., 2008; Ćurčić‐Blake et al., 2017; Diederen, Daalman, et al., 2012; Diederen, van Lutterveld, et al., 2012; Hugdahl, 2015; Upthegrove et al., 2016).

Numerous studies have investigated the pathological features of different subtypes of AHs, but relatively few studies have adopted a uniform index to investigate functional brain alterations among different subtypes of AHs. To address this gap in the literature, we conducted a study to investigate the common and distinct pathological brain features of four subtypes of AHs in AVs‐NSNMCS individuals. In this study, we adopted global functional connectivity density (gFCD) to investigate brain alterations across all samples. gFCD represents the number of connections between one voxel and other voxels in the whole brain. It can also be used as a quantitative index of neural activity in specific brain regions (Lang et al., 2015). A recent PET study indicated that gFCD is a potential biomarker of quantitative changes in glucose metabolism (Thompson et al., 2016). This research indicated that gFCD alterations reflect information communication capacity of the whole brain and can be used as a quality index to assess metabolism in specific brain regions (Qin, Xuan, Liu, Jiang, & Yu, 2015; Yin et al., 2017; Zhuo et al., 2014, 2017). In this study, we adopted the gFCD method to investigate functional brain alterations in different subtypes of AHs in AHs‐NSNMCS individuals. We hypothesized that patients with different AH subtypes would be associated with both common and distinct gFCD patterns.

## 2 METHODS

### 2.1 Samples

We recruited individuals with constant commanding and commenting auditory verbal hallucinations (CCCAVHs; *n* = 39), own thought auditory verbal hallucinations (OTAVHs; *n* = 42), nonverbal AHs (NVAHs; *n* = 46), or replay AVHs (RAVHs; *n* = 32), and 50 healthy controls to participate in this study by advertising recruitment from our database (Tianjin Mental Disorder Patients and High Risk Population Management Database, established from December 2012 to December 2016; Johns, Nazroo, Bebbington, & Kuipers, 2002; Yin et al., 2018). Inclusion criteria for the AVs‐NSNMCS‐individual sample were as follows: (a) Participants had abnormal perception that completely satisfied the AVH criteria (as per John et al.'s report), “Did you at any time hear voices saying quite a few words or sentences when there was no one around that might account for it?” or “Did you at any time hear some or single words but this or these words do not make sense when there was no one around that might account for it?” (*Note*: this is an operational definition referenced by Johns et al., 2002); (b) according to DSM‐IV Axis‐I and Axis‐II, participants did not completely satisfy any specific mental disorder diagnostic criteria, and SCI‐D was conducted by two senior psychiatrists with more than 10 years of experience; (c) participants did not satisfy any specific neurological disease diagnostic criteria; (d) participants did not receive any antipsychotic treatment for 2 weeks before participating in this study; and (e) participants had IQ > 80. Exclusion criteria were as follows: (a) Patients had other psychotic or affective disorders, mental retardation, alcohol dependence, drug dependence, organic brain lesions, or physical and neurological diseases; (b) history of unconsciousness for more than 5 min caused by any reason; (c) contraindications for MRI examination; (d) claustrophobia; and (e) IQ < 80. All participants were right‐handed. Healthy controls were distinguished by a professional psychiatrist using SCI‐D NP, which was also used by two professional psychiatrists. The subtypes of AHs were classified based on the definition provided by McCarthy‐Jones (McCarthy‐Jones et al., 2014). Written informed consent was obtained from all participants or their legal guardians. The Ethics Committee of Tianjin Anding Hospital and Wenzhou Seventh People's Hospital approved this study (TJMHPNGC‐2014‐001, ZS2017011).

### 2.2 Assessment of psychotic symptoms, cognition, and AH scores

All psychiatric assessments were performed by a trained psychiatrist for psychotic symptoms using Positive and Negative Symptom Scales (PANSS; Tibber et al., 2018). Global functioning was estimated using the GAF scale (Aas, Sonesson, & Torp, 2018). GAF was scored as the highest level of functioning over the past year, defined by the lowest score in social, psychological, or professional functioning. Psychiatric disorders in family members of the participants were quantified using the Family Interview for Genetic Studies (Wahab et al., 2015). We adopted the Auditory Hallucinations Rating Scale (AHRS; O'Brien et al., 2018) to assess the severity of AHs in AVs‐NSNMCS individuals. Cognitive assessments in this study adopted the MATRICS Consensus Cognitive Battery (MCCB; Holmén, Juuhl‐Langseth, Thormodsen, Melle, & Rund, 2010). Urine samples were obtained to screen for drug abuse (cannabis, amphetamine, cocaine, methadone, heroin, or other substances of abuse). Positive screening for any of these substances led to exclusion. Individuals with psychiatric disorders in family members also led to exclusion.

### 2.3 Functional MRI examination

Functional magnetic resonance imaging (fMRI) was performed using a GE Healthcare Discovery MR750 3T MRI system (General Electric) with an eight‐channel phased‐array head coil. Participants were instructed to lie supine, quieten their thoughts, and minimize head motion during the examination. The imaging parameters were as follows: 2,000‐ms repetition time (TR), 45‐ms echo time (TE), 32 slices, 4‐mm slice thickness, 0.5‐mm gap, 220 × 220 field of view (FOV), 64 × 64 acquisition matrix, and 90° flip angle. All scans were acquired with parallel imaging using SENSitivity Encoding (SENSE), with a SENSE factor of 2. Structural images were obtained with a high‐resolution three‐dimensional turbo‐fast echo T1‐weighted sequence with the following parameters: 8.2/3.2‐ms TR/TE, 188 slices, 1‐mm thickness, no gap, 256 × 256 FOV, 256 × 256 acquisition matrix, and 12° FA.

### 2.4 fMRI data preprocessing

Statistical Parametric Mapping 8 (SPM8; http://www.fil.ion.ucl.ac.uk/spm) was used to process resting‐state fMRI scans. To allow stabilization of the scanner and acclimation of patients to the environment, the first 10 scan volumes were discarded. The remaining volumes were corrected for slice timing and motion artefacts. fMRI data were within the allowable motion thresholds (translational and rotational motion <2 mm and 2°, respectively). Six of the motion parameters and average blood oxygen level‐dependent (BOLD) signals of the ventricles and white matter were removed. Next, framewise displacement (FD) was calculated, and data with specific‐volume FD > 0.5 were excluded from the study. The datasets were filtered with band‐pass frequencies ranging from 0.01 to 0.08 Hz. Individual structural images were coregistered to the mean functional image. The transformed structural images were coregistered to the Montreal Neurological Institute (MNI) space using linear registration. The motion‐corrected functional volumes were spatially normalized to the MNI space using parameters estimated during linear coregistration. Finally, the functional images were resampled into 3‐mm cubic voxels for further analysis.

### 2.5 gFCD calculation

The gFCD was calculated for each voxel using an in‐house Linux script (Waters, 2012). Functional connectivity between voxels was evaluated using Pearson's linear correlation with a correlation coefficient threshold of *R* > .6. gFCD calculations were limited to voxels within the cerebral gray matter mask. The gFCD for any given voxel (x0) was calculated as the total number of functional connections [*k*(x0)] between x0 and all other voxels using a growth algorithm. This procedure was repeated for all x0 voxels. The rsgFCD maps were spatially smoothed using a 6 × 6 × 6‐mm full‐width at half maximum Gaussian kernel. Each gFCD value was divided by the mean value from all included voxels to increase the normality of the distribution.

### 2.6 Statistical analysis

One‐way ANOVA was used to analyze sociodemographic information, severity of psychotic symptoms, or persistent time of AHs between groups of AHs‐NSNMCS individuals. Differences in gFCD among groups were tested using voxel‐wise one‐way analysis of covariance (ANCOVA), with age, sex, education level, GAF scores, and PANSS scores as covariates, followed by post hoc intergroup comparisons. Intergroup comparisons were conducted within a mask showing gFCD differences from the ANCOVA analysis. Multiple comparisons were corrected using the family‐wise error (FWE) method with a significance threshold of *p* < .05. Differences in gFCD between different AH subtypes were compared with *t* test. To investigate the relationship between gFCD and total AHRS score, a step‐wise multiple regression analysis was conducted; regions showing significant gFCD differences in AHs‐NSNMCS individuals were compared with the same regions in other groups. Given the importance of AH severity for neural correlations, we examined the correlation between gFCD and AHRS score in all samples with AHs, followed by FWE correction for multiple comparisons.

## 3 RESULTS

### 3.1 Sample demographic and clinical characteristics

Sociodemographic information of participants is depicted in Table 1. Gender, age, education level, cognitive scores, AH scores, and GAF scores were significantly different. PANSS scores remained significantly different among groups.

**Table 1:** Demographic and clinical characteristics of the samples

Variable	Healthy controls *N* = 50	CCCAVHs individuals *N* = 39	OTAVHs individuals *N* = 42	NVAHs individuals *N* = 46	RAVHs individuals *N* = 32	*F*/*t*	*p*
Gender, male/female	25/25	24/15	28/164	25/21	12/20	18.950	<.001
Age, years Mean (*SD*)	22.0 (2.5)	24.7 (3.2)	21.9 (1.8)	20.0 (1.5)	25.5 (4.8)	32.511	<.001
Education level, years Mean (*SD*)	15.4 (3.5)	12.0 (3.5)	14.0 (3.7)	10.0 (1.7)	16.0 (4.7)	39.000	<.001
PANSS scores Mean (*SD*)	32.5 (2.5)	35.2 (3.7)	34.1 (1.1)	36.3 (3.0)	38.0 (4.0)	11.100	<.001
Ahs scores Mean (*SD*)	N/A	20.8 (5.1)	15.4 (2.9)	18.5 (4.5)	28.0 (7.6)	25.810	<.001
GAF scores Mean (*SD*)	100.0 (0.0)	75.8 (10.2)	80.2 (9.8)	75.0 (12.0)	70.0 (10.5)	25.810	<.001
MCCB test							
TMT: part A	27.5 (9.8)	36.7 (7.5)	42.4 (8.9)	38.1 (10.0)	39.1 (7.0)	42.23	<.001
BACS symbol coding	69.9 (9.8)	48.2 (5.1)	50.0 (4.3)	54.0 (7.7)	55.0 (9.3)	28.231	<.001
HVLT‐R	30.0 (1.2)	26.8 (3.0)	25.0 (2.3)	27.5 (4.0)	25.7 (3.6)	17.890	<.001
WMS‐III spatial span	20.0 (2.0)	16.6 (3.5)	15.7 (4.5)	17.0 (2.1)	16.0 (3.0)	24.260	<.001
Letter‐number span	17.0 (1.9)	14.3 (3.3)	15.0 (2.2)	14.0 (2.9)	15.9 (2.0)	16.369	<.001
NAB mazes	22.7 (3.5)	14.9 (3.5)	17.8 (5.7)	16.4 (2.0)	15.9 (4.0)	16.480	<.001
BVMT‐R	28.5 (3.7)	20.5 (5.0)	22.7 (3.8)	19.7 (4.0)	22.1 (3.1)	12.211	<.001
Category fluency: animal naming	24.0 (4.8)	18.0 (2.1)	19.4 (2.2)	17.5 (3.5)	22.3 (5.0)	17.123	<.001
MSCEIT: managing emotions	92.0 (10.4)	79.5 (8.1)	82.5 (5.1)	75.5 (8.0)	79.5 (3.5)	25.221	<.001
CPT‐IP DPrime	2.3 (0.3)	1.6 (0.7)	1.5 (0.4)	1.8 (0.6)	2.0 (0.2)	10.473	<.001

### 3.2 gFCD differences compared to healthy controls

**Figure 1:**
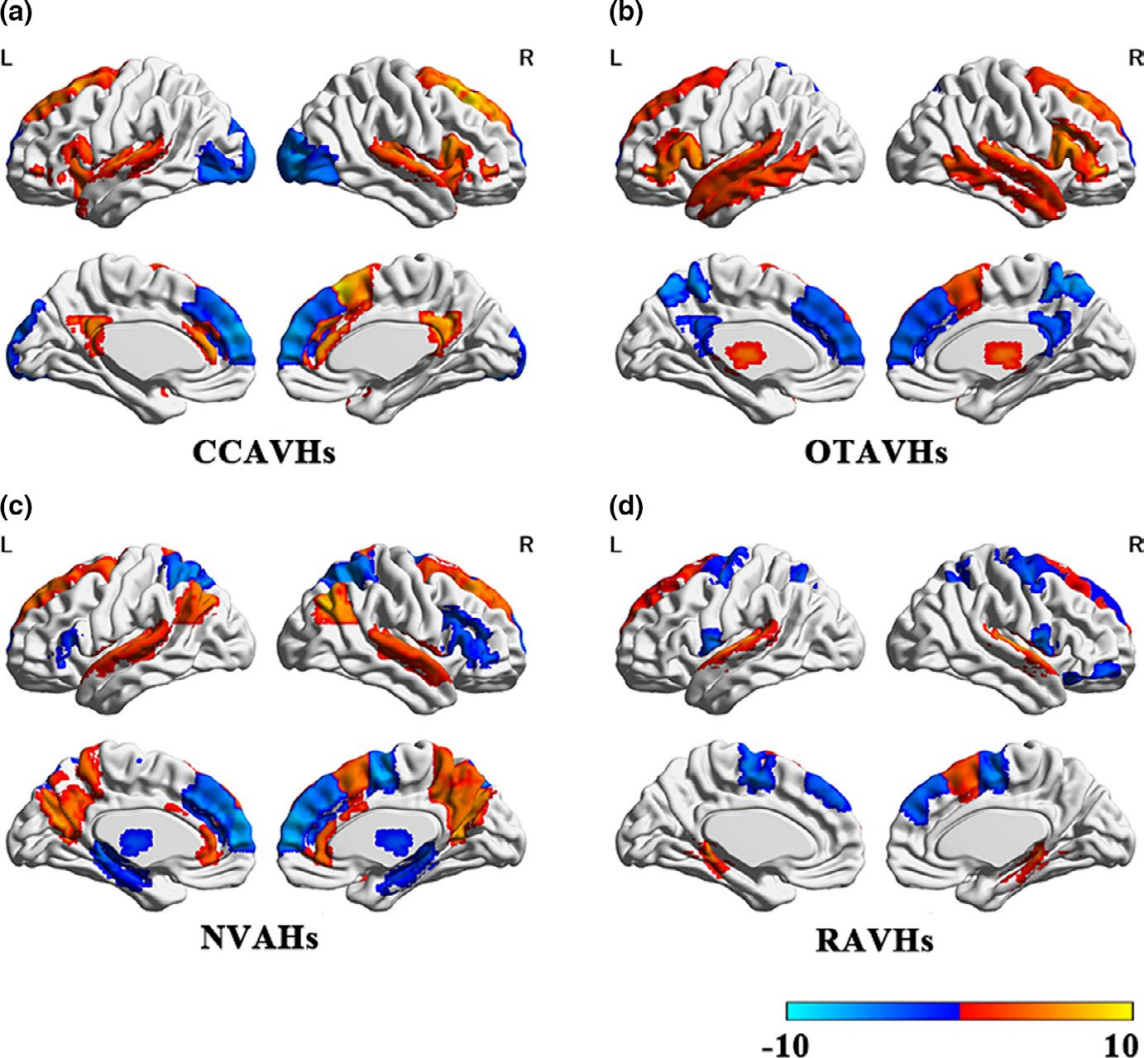
Global functional connectivity density (gFCD) alterations among AVs‐NSNMCS individuals compared to healthy controls

Compared to healthy controls, samples with CCCAVHs demonstrated increased gFCD in the bilateral Broca's area, bilateral superior temporal gyrus, bilateral supramarginal gyrus, bilateral insula, bilateral anterior cingulate gyrus, mesial frontal lobe, and bilateral posterior cingulate gyrus. Decreased gFCD was observed in the bilateral occipital lobule, bilateral medial prefrontal cortex, precentral gyrus, postcentral gyrus, and lingual gyrus. Compared to healthy controls, samples with OTAVHs demonstrated increased gFCD in the bilateral Broca's area, bilateral superior temporal gyrus, mesial frontal lobe, pulvinar thalamus, bilateral superior‐middle temporal, inferior frontal gyrus, and bilateral thalamus. Decreased gFCD was observed in the bilateral medial prefrontal cortex, bilateral posterior parietal cortex, and bilateral posterior cingulate cortex. Compared to healthy controls, samples with NVAHs demonstrated increased gFCD in the bilateral superior temporal gyrus, mesial frontal lobe, bilateral anterior and posterior cingulate gyri, bilateral precuneus, and bilateral angular gyrus. Decreased gFCD was observed in the bilateral medial prefrontal cortex, bilateral inferior frontal gyrus, bilateral parietal lobe, bilateral hippocampus, and bilateral thalamus. Compared to healthy controls, samples with RAVHs demonstrated increased gFCD in the bilateral superior temporal gyrus, mesial frontal lobe, bilateral hippocampus, and cerebellum. Decreased gFCD was observed in the bilateral intraparietal gyrus, bilateral medial precentral gyrus, bilateral superior frontal gyrus, and right ventromedial/orbitofrontal cortex (OFC; Figure 1, Peak value in Table 2).

**Table 2:** The areas and the coordinates of the peak value of the images

	Peak MNI coordinate		
	*x*	*y*	*z*
CCAVHs			
Increase			
Insula	−33	12	6
	36	24	0
PCC	6	−45	33
Temporal_sup	57	−27	15
	−59	−21	12
mFC	3	27	51
	−24	15	57
Decrease			
Occipital lobe	−21	−93	6
	30	−87	3
mPFC	0	51	6
OTAVHs			
Increase			
Frontal_Inf	−42	45	0
	45	18	27
Temporal_sup	48	−18	12
	−36	−27	12
Thalamus	18	−24	−3
	6	−12	9
	24	33	45
Decrease			
PCC	6	−51	21
Precuneus	9	−60	60
mPFC	0	49	7
NVAHs			
Increase			
ACC	0	42	3
Precuneus	33	−60	36
Temporal_sup	57	−27	15
	−36	−27	12
mFC	24	33	45
	−9	45	42
Decrease			
Thalamus	15	−39	0
Frontal_Inf	36	15	6
	−36	12	6
Parietal lobe	−24	−69	48
	30	−54	54
mPFC	−2	50	6
	3	−3	51
RAVHs			
Increase			
Hipp	15	−39	0
	−12	−39	2
Temporal_sup	51	−18	12
	−57	−21	11
mFC	24	30	48
	−24	−9	63
Decrease			
Frontal_sup	0	−12	54
IntraParietal	−30	−60	42
	33	−57	42
Right Frontal_Orb	36	39	−9
OTAVHs versus CCAVHs			
Increase			
Hipp	27	−15	−18
	−24	−9	−15
Precentral	45	−15	60
	−24	−27	63
Thalamus	3	−9	9
Left Frontal_inf	−42	45	0
Decrease			
Cingulum_Mid	6	−45	36
Right cerebullum	12	−66	−45
NVAHs versus CCAVHs			
Increase			
ACC	−3	39	12
Angular	33	−57	36
Parietal_sup	−33	−57	51
	−24	−69	48
Preceneus	12	−54	18
	−12	−57	20
Decrease			
Frontal pole	3	45	−6
Fusiform gyrus	−24	−66	−12
	23	−66	−14
Hipp	18	−30	−6
	−12	−39	0
Posterior parietal cortex	9	−60	60
Thalamus	−9	−18	9
Putamen	33	−3	3
	−33	−6	9
RAVHs versus CCAVHs			
Increase			
ACC	6	42	−6
Hipp	−12	−3	12
	12	−38	0
Insula	36	0	−3
	−36	−1	−6
PCC	9	−39	27
SupraMarginal	63	−21	30
	−57	−27	39
ParaHipp	−15	−12	−30
	18	−6	−30
Decrease			
Lingual gyrus	−12	−90	−6
Occipital lobe	−30	−87	9
	33	−78	12
Right Frontal_orb	33	39	−9
Subgenual cingulate cortex	6	39	18
Common			
Increase			
Temporal_sup	−36	−27	12
	48	−18	12
Mesial frontal cortex	3	−9	54
Decrease			
mPFC	3	57	21
Distinct			
CCAVHs			
Insula	−33	12	6
	36	24	0
OTAVHs			
Thalumus	18	−24	−3
	6	−12	9
NVAHs			
Precuneus	9	−54	18
RAVHs			
Hipp	15	−39	0
	−12	−39	2

### 3.3 gFCD differences compared to samples with CCCAVHS

**Figure 2:**
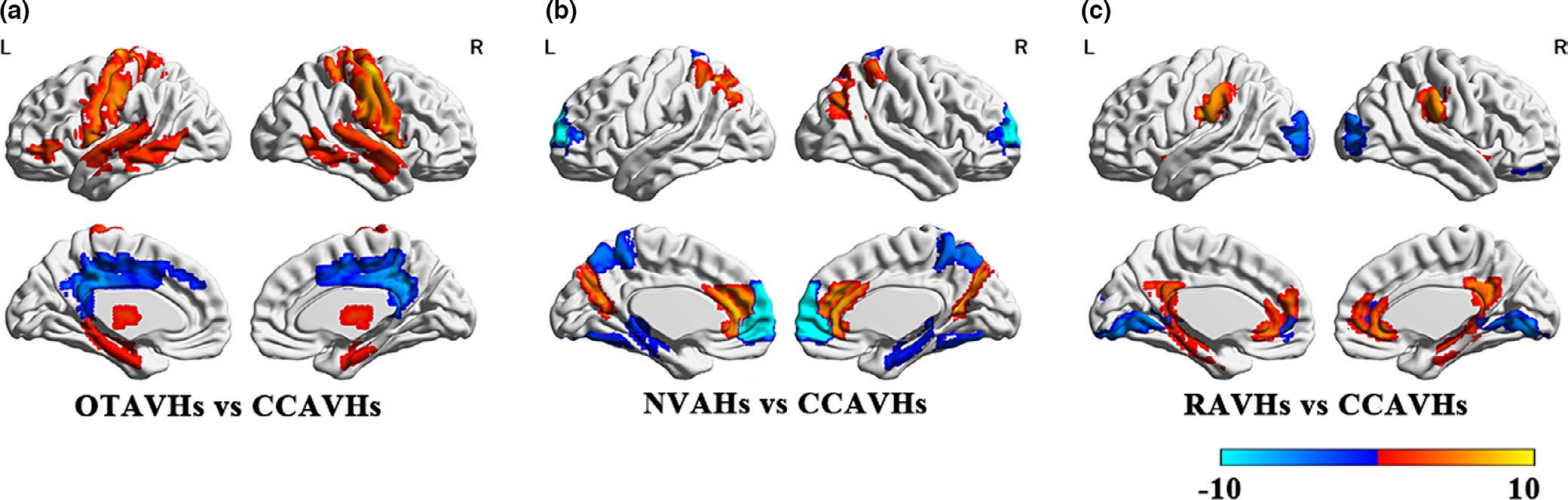
Global functional connectivity density (gFCD) alterations among three different subtypes AVs‐NSNMCS individuals compared to CCCAVHs samples

Compared to samples with CCCAVHS, samples with OTAVHs demonstrated increased gFCD in the bilateral thalamus, bilateral hippocampus, bilateral precentral gyrus, left inferior frontal gyrus, and sensorimotor cortex. Decreased gFCD was observed in the bilateral posterior cingulate cortex, bilateral middle cingulate cortex, and left cerebellum. Compared to samples with CCCAVHS, samples with NVAHs demonstrated increased gFCD in the bilateral precuneus, bilateral angular gyrus, bilateral superior parietal lobule, and bilateral anterior cingulate cortex. Decreased gFCD was observed in the bilateral posterior parietal lobe, bilateral hippocampus, bilateral thalamus, bilateral frontal pole, putamen, and fusiform gyrus. Compared to samples with CCCAVHS, samples with RAVHs demonstrated increased gFCD in the bilateral hippocampus, bilateral thalamus, bilateral anterior cingulate cortex, posterior cingulate cortex, bilateral insular cortex, bilateral supramarginal gyrus, and bilateral parahippocampal gyrus. Decreased gFCD was observed in the right ventromedial/OFC, bilateral occipital lobule, and subgenual cingulate cortex (sgCC; Figure 2, Peak value in Table 2).

### 3.4 Association of gFCD with AH severity

No significant correlation between gFCD and AH severity (ARHS total score and ARHS frequency) was observed in AVs‐NSNMCS individuals.

## 4 DISCUSSION

In this study, we firstly proposed an operational definition of AVs‐NSNMCS individuals that replaces previous definitions such as “healthy individuals with auditory verbal hallucinations,” “non‐psychotic individuals with voice hearing,” “non‐psychotic adults with frequent auditory verbal hallucinations,” and “healthy individuals prone to auditory hallucinations.” In previous studies, operational definitions of different samples differed, and enrolment criteria of samples emphasized items such as “hearing voices in the absence of objective stimuli” and “did not satisfy any specific mental disorders and neurological diseases.” However, these samples should not be considered healthy, as many studies suggested that these samples have global functional deficits and impaired cognition. Moreover, previous studies confirmed that these samples are at high risk of developing psychosis or may need medical intervention. Hence, in this study, we standardized these samples as AVs‐NSNMCS individuals. We emphasized the features of “hearing voices in the absence of objective stimuli” and “did not currently satisfy any specific mental disorders or neurological diseases.” We propose that this definition will assist investigation and facilitate therapeutic interventions to reduce the rate of conversion to psychosis or deterioration of global function. Our clinical assessment demonstrated that GAF scores and MCCB performance of these samples were impaired relative to that of healthy controls. These findings support our “AVs‐NSNMCS‐individuals” definition from clinical features. Our proposed definition addresses a gap in the literature. These samples are at high risk of psychosis, for which many interventions have been proposed, including antipsychotic agents. However, if we intervention healthy individuals may be caused accountability, after all, only professional psychiatrist know these samples are the high‐risk populations needed early intervention.

**Figure 3:**
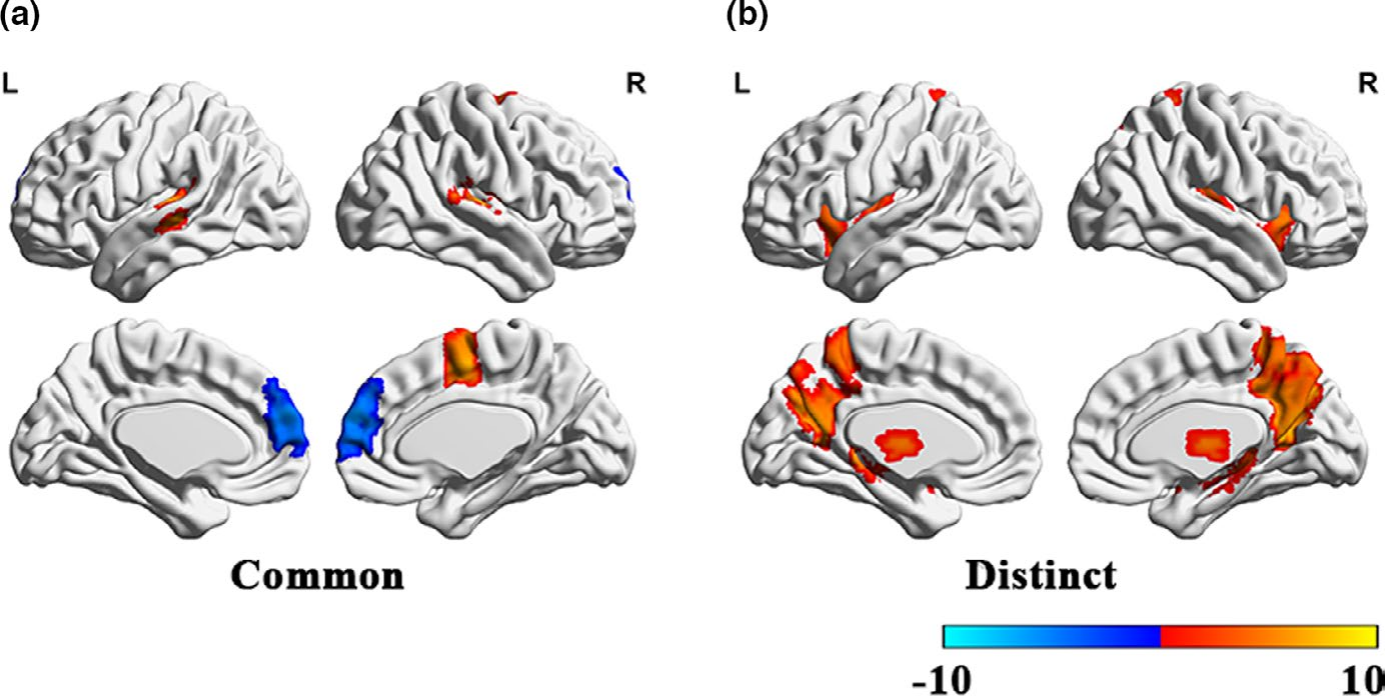
The common and distinct global functional connectivity density (gFCD) alterations across four subtypes of AVs‐NSNMCS individuals

The second important finding of our study was that we observed four subtypes of AVs‐NSNMCS individuals with common and distinct functional brain activity (Figure 3, Peak value in Table 2). The common substrates across four subtypes of AVs‐NSNMCS individuals were increased gFCD in the bilateral superior temporal gyrus and mesial frontal lobe, and decreased gFCD in the bilateral medial prefrontal cortex. Distinct functional brain patterns were increased gFCD in the bilateral insula in CCCAVH individuals, increased gFCD located in the bilateral thalamus in OTAVH individuals, increased gFCD in the bilateral precuneus in NVAH individuals, and increased gFCD in the bilateral hippocampus in RAVHS individuals. Commonalities in brain alterations across AVs‐NSNMCS individuals indicated that dysfunction of the bilateral superior temporal gyrus and bilateral medial prefrontal cortex may be common pathological features of AHs. Hyperactivity in the bilateral superior temporal gyrus and hypoactivity in the bilateral medial prefrontal cortex may form the neural basis of AHs. Many hypotheses regarding AHs propose that hyperactivity of the superior temporal gyrus and mesial frontal lobe may underlie the generation of AHs, and hypoactivity of the prefrontal lobe attenuates inhibition of abnormal hyperactivity in the superior temporal gyrus. This phenomenon may cause “false voice”‐related neural activity to become the “reality experience” of individuals with AHs and thereby cause AHs. Our findings support abnormal reciprocal actions between the superior temporal gyrus, mesial frontal lobe, and prefrontal lobe in AH generations, consistent with many hypotheses on AHs. The abnormal reciprocal action between the superior temporal gyrus and prefrontal lobe may be a common circuit in different subtypes of AHs. Our findings also indicated that this common pathway may be a treatment target for AH intervention.

Compared to individuals with CCCAVHs individuals, those with OTAVHs, NVAHs, or RAVHs exhibited different brain activity patterns. Individuals with OTAVH demonstrated dysfunction in the bilateral thalamus, bilateral hippocampus, bilateral precentral gyrus, left inferior frontal gyrus, sensorimotor cortex, bilateral posterior cingulate cortex, bilateral middle cingulate cortex, and left cerebellum. These regions are pivotal components of the limbic system, which is related to mood processing, attention, and memory. In NVAH individuals, dysfunction was observed in the bilateral precuneus, bilateral angular gyrus, bilateral superior parietal lobule, bilateral anterior cingulate cortex, bilateral posterior parietal lobe, bilateral hippocampus, bilateral thalamus, bilateral frontal pole, putamen, and fusiform gyrus. These findings indicated abnormal reciprocal actions of hyperactivity in the default mode network and hypoactivity in mood and memory processing circuits. Executive‐related circuits in the frontal lobe are the neural basis in NVAH individuals, supporting the hypothesis that the default mode network is involved in AH generation (Holmén et al., 2010). In individuals with RAVHs, dysfunction was observed in the bilateral hippocampus, bilateral thalamus, bilateral anterior cingulate cortex, posterior cingulate cortex, bilateral insula cortex, bilateral parahippocampal gyrus, right ventromedial/OFC, bilateral occipital lobule, sgCC, and lingual gyrus. These regions are the components of the salience network, mood processing, and memory processing circuits. Abnormal reciprocal action in these regions may cause reality distortion of perception, inducing unnecessary thoughts or perceptions, which become AHs. These findings are consistent with the hypothesis that “memory and thought intrusion, and deficits in reality monitoring” are the neural basis of AHs. CCCAVH is the pivotal symptom, usually accompanied by the greatest risks of suicide and violence, and placed in the first class of symptoms of schizophrenia (Alderson‐Day et al., 2015; Harkavy‐Friedman et al., 2003; McCarthy‐Jones et al., 2014). Although it can occur in healthy individuals, most individuals with CCCAVHs are or become psychotic (Daalman et al., 2016; Sommer et al., 2010). Hence, characterization of the brain features of CCCAHVs in healthy individuals is more important than the characterization of other AH features. Hence, we only compared CCCAVH individuals to healthy controls in the present study; thus, we were unable to describe specific alterations of CCCAVHs relative to those of other AH subtypes. CCCAVHs were related to disturbances in reciprocal action among bilateral Broca's area, bilateral superior temporal gyrus, bilateral insular, bilateral anterior cingulated gyrus, mesial frontal lobe, bilateral posterior cingulate gyrus, bilateral supramarginal gyrus, precentral gyrus, postcentral gyrus, bilateral occipital lobule, and bilateral medial prefrontal cortex. These regions are the core components of the executive control network and primary auditory cortex; abnormal reciprocal activity between these regions supports the hypothesis that the interaction between speech generation and speech perception processes gives rise to the phenomenological experience of sound in the absence of external stimuli.

In summary, our findings converge with those of previous studies, supporting the view that pathological features in individuals with CCCAVHs include abnormal reciprocal actions among the main components of the executive attentional network, speech generation, and speech perception circuits. CCCAVHs pathological features support the bottom‐up model hypothesis of AHs (Zmigrod, Garrison, Carr, & Simons, 2016). The pathological features of OTAVHs individuals include abnormal reciprocal action among limbic systems, mood processing, attention processing, and memory processing. These findings support the hypothesis that misattribution deficits are the basis of AHs (Northoff, 2014). The pathological features of NVAHs individuals highlight abnormal reciprocal action among the bilateral precuneus, bilateral angular gyrus, bilateral superior parietal lobule, bilateral anterior cingulate cortex, bilateral posterior parietal lobe, bilateral hippocampus, bilateral thalamus, bilateral frontal pole, putamen, and fusiform gyrus. These regions are key components of the default mode network, memory processing, and executive processing. These findings support the hypothesis that emphasizes default mode network instability and abnormal activity in anterior–posterior networks as the basis of AHs (Alderson‐Day et al., 2015). For RAHS individuals, pathological features were observed in the bilateral hippocampus, bilateral thalamus, bilateral anterior cingulate cortex, posterior cingulate cortex, bilateral insular cortex, bilateral parahippocampal gyrus, right ventromedial/OFC, bilateral occipital lobule, sgCC, and lingual gyrus. These regions are key components of the salience network, mood regulation network, and memory processing network. These findings support the hypothesis that the SN is necessary for initiating and modifying sensory information and actions (Menon & Uddin, 2010), and abnormal activity in the insula is essential for the generation of AHs via evaluation of stimuli and attribution of salience. Aberrant activity within the claustrum–insula complex may be involved in impaired proximal salience appraisal leading to AVH, with compensatory activity in other areas, including the auditory network and areas involved in auditory processing, language, and memory, as well as areas involved in network dysconnectivity in predictive coding such as the cerebellum (Cierpka et al., 2017; Habas et al., 2009). In sum, CCCAVHs support the bottom‐up model hypothesis of AHs, OTAVHs support the monitoring deficit and instable memory hypothesis of AHs, NVAHs support the hypothesis of DMN instability and abnormal activity in anterior–posterior networks as the basis of AHs, and RAHS support the hypothesis that the SN is necessary to initiate and modify sensory information and actions. Notably, we observed that functional activity was located in bilaterally symmetrical regions, supporting the hypothesis of interhemispheric miscommunication and hybrid models of AVH. Notably, this feature was common across different subtypes of AHs.

### 4.1 Limitations

Three limitations of this study should be considered. First, we selected only samples without specific mental disorders; this was a priority of our study to avoid confounding factors such as therapeutics and other psychotic symptoms. Previous hypotheses of AHs were established mainly on studies of schizophrenia and other mental disorders. Although our findings support the above hypotheses, further studies are required for clarification. Second, cross‐sectional studies do not provide strong evidence for differences between subtypes of AHs. Long‐term studies on large samples will help to describe the dynamic trajectories of neural alterations and trajectory of AH symptoms to elucidate effective intervention strategies.

## 5 CONCLUSIONS

To our knowledge, this report is the first to describe gFCD alterations in AVs‐NSNMCS individuals. The important findings of this study were that different subtypes of AHs were underpinned by distinct functional brain activity patterns. The common and distinct gFCD alterations among these four AHs subtypes were observed mainly in components of the frontoparietal network, default mode network, salience network, central executive network, and memory network. These findings indicated that aberrant functional activity and metabolism in the abovementioned networks may be involved in the occurrence of AHs. Our findings provide evidence to support different hypotheses of AHs based on distinct gFCD alterations in specific subtypes of AHs.

## CONFLICT OF INTEREST

The authors declared no conflict of interest.

## Data Availability

The datasets generated and analyzed during the present study are available from the corresponding author on reasonable request.
